# Marking dad’s centromeres: maintaining CENP-A in sperm

**DOI:** 10.1007/s10577-025-09766-2

**Published:** 2025-04-26

**Authors:** Miriama Štiavnická, Rachel S. Keegan, Elaine M. Dunleavy

**Affiliations:** Centre for Chromosome Biology, Biomedical Science Building, University of Galway, Galway, H91W2TY Ireland

**Keywords:** CENP-A, Centromere, Sperm, Histone, Gamete, Male fertility

## Abstract

During spermiogenesis, histones are removed from most genomic loci and are replaced by protamines in mature sperm nuclei. Yet, centromeres appear resistant to this process. We review the experimental evidence that the centromeric histone CENP-A is maintained in mature sperm nuclei, comparing human, bovine, mouse and fly species. We also recall how the detection of centromeres in mature sperm nuclei in the 1990’s contributed to the isolation of the CENP-A protein and the eventual cloning of the human CENP-A gene. Further, based on more recent genetic studies carried out in flies and in mice, we discuss the inheritance and functional importance of paternal CENP-A and how it is complemented by maternal CENP-A to give rise to a healthy embryo. Finally, we raise some unanswered questions regarding the exclusive maintenance of CENP-A on sperm, the organisation of sperm centromeric chromatin and its importance for fertility and early embryo development.

## Introduction

Centromeres are the essential regions on chromosomes where microtubules attach during cell division, facilitating the correct distribution of genetic material to daughter cells. These regions are embedded within tandemly repeated DNA sequences, which in humans are 171 base pair long and are organised into higher-order repeats (Miga and Alexandrov 2021; Altemose et al. 2022a). However, this DNA sequence is not sufficient or necessary to define centromere location. Instead, the centromere is uniquely marked by the essential presence of Centromeric Protein A (CENP-A), a variant of histone H3 (McKinley and Cheeseman 2015), which replaces histone H3 in a small percentage of nucleosomes at the centromere (Bodor et al. 2014).

In somatic cells, CENP-A functions in conjunction with the constitutive centromere-associated network (CCAN) (Foltz et al. 2006; Yatskevich et al. 2023), which includes the well-characterised binding partner CENP-C. In addition, CENP-B binds to both CENP-A and centromeric DNA (Dumont and Fachinetti 2017). The functions of these proteins are relatively well understood throughout the cell cycle, particularly during mitosis (Kixmoeller et al. 2020). However, the role of CENP-A, CENP-B and CCAN components in germ cells undergoing meiosis, and in the resulting gametes, is not well studied. Mature sperm is particularly interesting in this context, because their nuclei undergo extensive chromatin remodelling, replacing most histones with protamines during the highly conserved process of spermiogenesis. After protamination, only 1–10% of histones are maintained in the chromatin of mature mammalian sperm (Moritz and Hammoud 2022; Gaspa-Toneu and Peters 2023). Key studies performed on mammalian sperm during the 1980’s first indicated that CENP-A was detectable in protamine-bound nuclei, evading this global chromatin remodelling event (Brinkley et al. 1986; Del Mazo et al. 1986; Sumner 1987). However, technical challenges related to the compaction of sperm chromatin slowed down progress in this area and knowledge about the maintenance of specific centromere proteins on mature sperm is still quite limited, even after 40 years.

Much of what is known about sperm centromere function stems from genetically tractable model systems, such as the fruit fly *Drosophila melanogaster* (Dunleavy et al. 2012; Raychaudhuri et al. 2012) and more recently mouse (Das et al. 2022). Similar investigations are lacking in humans as germline tissues and cells are generally hard to access. However, advances in genome editing and the availability of complete centromere genome assemblies are now enabling such investigations in other mammals and diverse species. This review aims to consolidate existing knowledge regarding CENP-A maintenance and function in mature sperm across flies and mammals and to provide perspectives for future research.

## The discovery of CENP-A – how sperm played its part

**Fig. 1:**
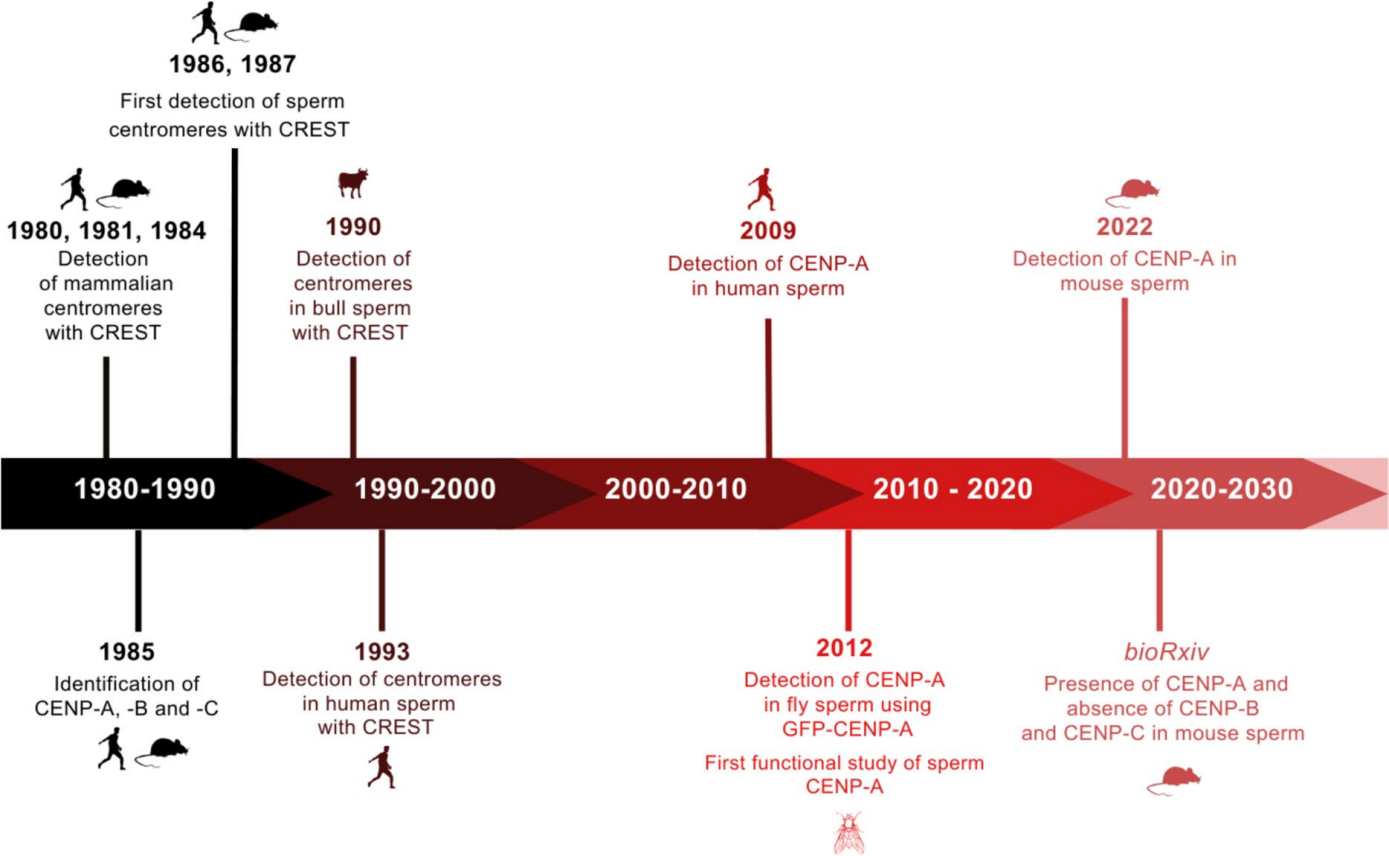
Chronological overview of the major events that led to the CENP-A localisation in mature sperm. The timeline starts with studies focused on the identification of antigens recognised by CREST serum in the 1980’s and continues with the detection of centromeres in decondensed mature sperm using CREST in the early 1990’s (Moroi et al. 1980, 1981; Brenner et al. 1981; Earnshaw et al. 1984; Earnshaw and Rothfield 1985; Brinkley et al. 1986; Del Mazo et al. 1986, 1987; Palmer et al. 1990; Zalensky et al. 1993). Since the late 2000’s, CENP-A was detected using purified antibodies from CREST serum, monoclonal anti-CENP-A antibodies or genetically tagged versions of CENP-A (Mudrak et al. 2009; Dunleavy et al. 2012; Raychaudhuri et al. 2012; Das et al. 2022; Manske et al. 2022). The first functional study showing the importance of sperm CENP-A for embryo development in flies was in 2012 (Raychaudhuri et al. 2012)

To review the current understanding of CENP-A maintenance and function in sperm (Fig. 1), it is important to revisit studies on the autoimmune disorder systemic scleroderma (Calcinosis, Raynaud phenomenon, Esophageal dysmotility, Sclerodactyly, and Telangiectasia or CREST syndrome). Early investigations using serum from CREST syndrome patients first indicated it contained antibodies recognising different antigens within the cell nucleus, in particular centromeres (Moroi et al. 1980, 1981; Brenner et al. 1981). In 1980, blood sera from 32 patients with CREST syndrome were used to stain a variety of mouse tissues and human cell lines. Across various stages of mitosis, the authors observed foci that were associated with condensed chromosomes. To verify this localisation, CREST serum was incubated with chromosomal spreads, with almost a third of the sera recognising the primary chromosomal constriction indicative of centromeres (Moroi et al. 1980). Electron microscopy extended these findings, showing foci localised to mitotic kinetochores and, in interphase cells, near the nuclear envelope and nucleoli (Moroi et al. 1981). Centromeric localisation was further confirmed by Earnshaw et al. (1984) on chromosomes from human HeLa S3 cells stained with CREST serum. Earnshaw and Rothfield (1985) went on to identify distinct antigens recognised by CREST serum using western analysis. Three dominant proteins, with molecular weights of 17, 80, and 140 kDa respectively, were subsequently identified and named CENP-A, CENP-B, and CENP-C (Earnshaw and Rothfield 1985).

Around the same time, CREST staining of mouse testes was also performed. Different distributions of centromere foci were initially noted in various stages of spermatogenesis, as well as an unexpected weakening of centromere staining through progression of meiosis (Del Mazo et al. 1986). However, it was a series of experiments performed using mature bovine sperm by Palmer and colleagues (Palmer et al. 1990, 1991) that led to the isolation of the CENP-A protein. Significantly, bovine sperm was selected as the source material for these experiments as it was available in abundant quantities and its lack of canonical histones enabled homogenous purification of CENP-A. These experiments not only showed that CENP-A was present in the nuclear fraction, but that it co-eluted with histones. Following biochemical purification, chymotrypsin digestion of CENP-A into peptides revealed a partial alignment with bovine histone H3, showing over 50% sequence identity (Palmer et al. 1990, 1991). Based on this bovine CENP-A sequence, the human sequence was first cloned and characterised as a centromere-specific histone H3 variant by (Sullivan et al. 1994).

## CENP-A at sperm centromeres: a lone survivor?

Given that early studies identifying sperm centromeres were carried out using human CREST serum, capable of recognising CENP-A, -B and -C, the exclusive retention of CENP-A on sperm was not directly assessed in cell biology studies performed at that time. Initially, staining of mouse testes did not detect a CREST signal in mature sperm (Brinkley et al. 1986; Del Mazo et al. 1986) suggesting that centromere proteins are lost during spermiogenesis. However, subsequent studies (del Mazo et al. 1987; Palmer et al. 1990; Zalensky et al. 1993) instead indicated that centromeres were not detected by the immunofluorescent method due to a high level of nuclear compaction in mature sperm. To overcome this compaction, sperm decondensation protocols were developed utilising: i) detergents to permeabilise the nuclear membrane, ii) reducing reagents to disrupt disulphide bonds between cysteine rich protamines, and iii) heparin or other polyanions to deplete protamines from DNA through charge modification (Jager et al. 1990; Cheng et al. 2009).

**Fig. 2:**
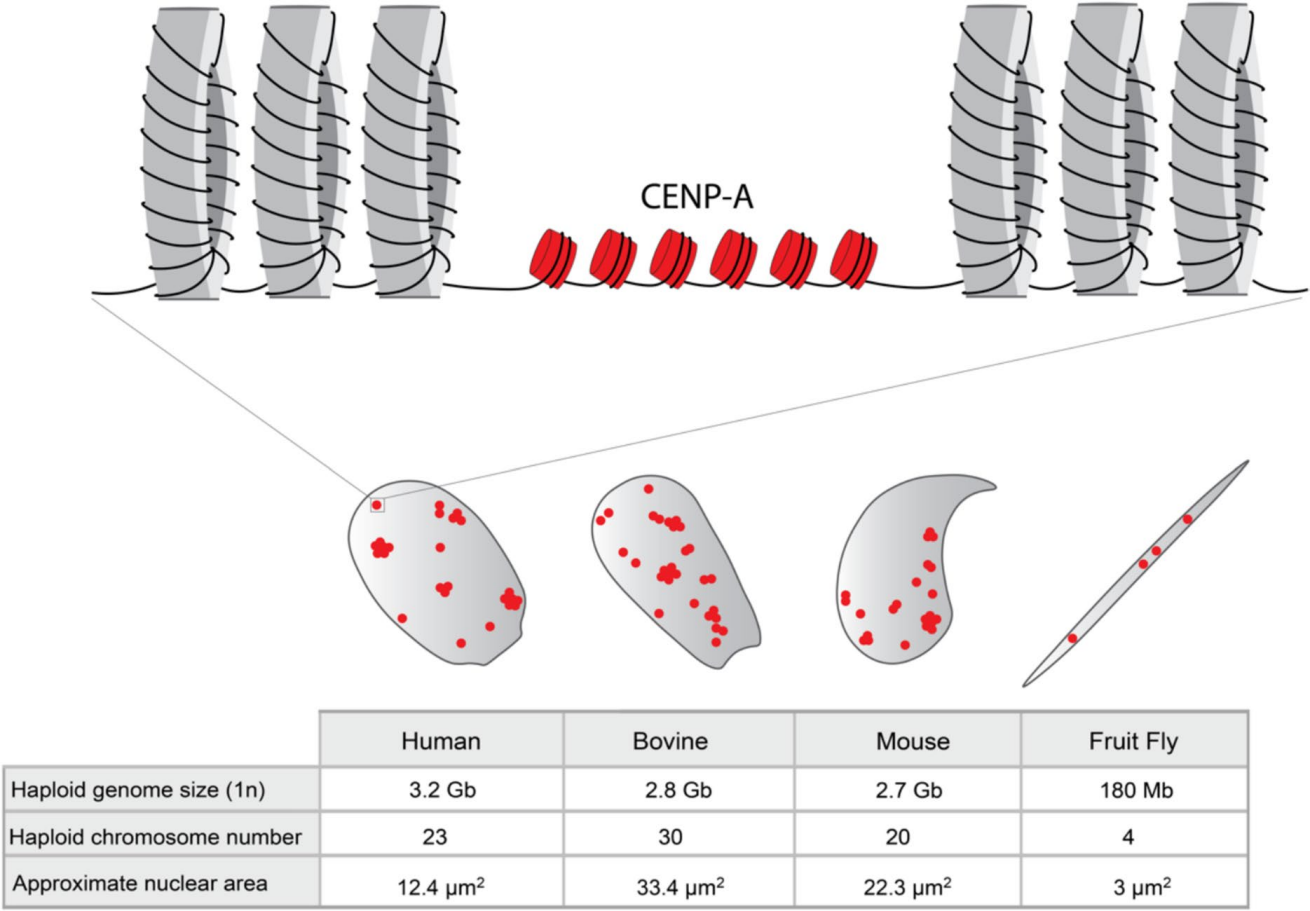
Chromatin packaging and centromere distribution in mature sperm nuclei. Schematic outlining the distribution of centromeres in the mature sperm nuclei of human, bovine, mouse and fruit fly (not to scale) (del Mazo et al. 1987; Palmer et al. 1991; Mudrak et al. 2009; Dunleavy et al. 2012; Raychaudhuri et al. 2012; Das et al. 2022). During spermiogenesis, 90% to 99% histones are removed, and the haploid genome is wrapped tightly around large protamine toroids. CENP-A (red foci) is retained in the mature sperm after histone to protamine exchange, although the precise composition and organisation of centromeric chromatin in this context is currently unknown (Del Mazo et al. 1986; Palmer et al. 1990; Zalensky et al. 1993). Mammalian centromeric foci cluster forming chromocenters, whereas fruit fly (*Drosophila melanogaster*) centromeres are distributed along the narrow needle-like nucleus (Del Mazo et al. 1986; Palmer et al. 1990; Zalensky et al. 1993). Also shown are haploid genome size, haploid chromosome number and approximate sperm nuclear area based on morphology and DNA staining for each species (Yániz et al. 2016; Li et al. 2023)

CREST serum was first applied to decondensed mouse sperm nuclei in the late 1980’s (del Mazo et al. 1987). The authors identified that centromere foci were located mainly at the periphery of the hooked-shaped mouse sperm nucleus (Fig. 2). They also noted that the number of foci did not correlate with the haploid chromosome number in mouse sperm, which was one of the first indicators of centromere clustering. Immunoblotting of the mouse sperm nuclear fraction with human CREST serum revealed a protein with a molecular weight of 18 kDa, representing CENP-A, while CENP-B and -C were not detected. Given that immunoblotting of somatic mouse tissues (thymus and liver) also solely identified CENP-A, unlike human lymphoma cells where CENP-B and CENP-C were recognised (del Mazo et al. 1987), it is possible that CREST serum did not recognise murine CENP-B or -C (del Mazo et al. 1987). It took almost 20 years before CENP-A staining of decondensed mature sperm was confirmed with an antibody (Champroux et al. 2018; Das et al. 2022). Very recently, CENP-A maintenance on sperm has been revisited using CRISPR/Cas9 to tag endogenous *Cenpa* with the red fluorescent protein mScarlet-I (Manske et al. 2022). In this study, the authors directly visualise endogenous C*enpa*^*mScarlet*^ foci in elongated sperm in mouse testes. Furthermore, they report for the first time the exclusive presence of CENP-A and absence of CENP-B and CENP-C in mature sperm by western analysis. In bovine sperm, the first localisation of centromeres was performed by Palmer et al. (1990) through immunofluorescent staining of decondensed nuclei with human CREST serum. Comparable to mouse, neither CENP-B nor CENP-C were detected by western blotting performed on bovine sperm (Palmer et al. 1990).

In the case of human, CREST serum was first applied to decondensed sperm nuclei in the 1980’s (Sumner 1987; Zalensky et al. 1993). Follow-up studies showed that human sperm displayed centromere foci dispersed across the nuclei (Zalensky et al. 1993) like the distribution observed in bull sperm (Palmer et al. 1990). In the late 2000’s, localisation of CENP-A in human sperm was confirmed by purified CREST serum specifically recognising CENP-A (Mudrak et al. 2009). For these experiments, sperm nuclei were incubated with extracts from oocytes of *Xenopus laevis* mimicking decondensation that naturally occurs at fertilisation. The authors observed that prolonged incubation with egg extracts led to the dissociation of CENP-A from centromeric satellite DNA sequences. While CENP-A moved toward nuclear edges, satellites remained organised in chromocenters. It is possible that similar organisational changes in the sperm nucleus may be happening in the zygote after fertilization.

## Sperm CENP-A inheritance and function: Insights from flies

Insights into CENP-A inheritance and the functional significance of its maintenance on sperm first emerged over decade ago from studies in the fruit fly *D. melanogaster.* In this animal, CENP-A is known to be the only centromeric protein to survive histone to protamine exchange in mature sperm (Dunleavy et al. 2012; Raychaudhuri et al. 2012). Limitations of immunofluorescence in sperm nuclei were overcome using a transgenic fly line expressing an EGFP-tagged CENP-A, which permitted the observation of distinct centromeric foci in mature sperm (Dunleavy et al. 2012; Raychaudhuri et al. 2012)(Fig. 2).

To address the inheritance pattern of sperm CENP-A, Raychaudhuri and colleagues tracked CENP-A-EGFP localisation after fertilisation (Raychaudhuri et al. 2012). They made two key observations. First, they observed that paternal CENP-A is detectable in the male pronucleus. Therefore, it is stably maintained throughout the reversal of protamine exchange and chromatin remodelling events that take place after fertilisation. Second, they observed that the paternal CENP-A is progressively replaced with maternally translated CENP-A. Taken together, these experiments provided the first evidence that paternal CENP-A is inherited and that it is diluted by maternal CENP-A before zygotic genome activation (ZGA).

To address the functional importance of paternally inherited CENP-A, the same authors used the deGradFP system to deplete GFP-tagged CENP-A from mature sperm. Remarkably, the authors found that depletion of sperm CENP-A to an undetectable level resulted in embryonic lethality upon fertilisation. This lethality arose from a failure to integrate the paternal chromosomes into the gonomeric spindle in the first embryonic division. In an additional experiment, CENP-A levels were reduced to approximately 50% in mature sperm using the GAL4-UAS system to deplete CENP-A under the control of a germline-specific driver. Reduced CENP-A levels were observed in the embryos produced from sperm carrying this low level of CENP-A. Furthermore, the weakened centromeres were quantitatively maintained beyond embryogenesis in the imaginal discs of third instar larvae and in the germline of adult male progeny. Thus, it seems that maternal stores cannot replenish a paternal deficit inherited from CENP-A-depleted sperm, and that CENP-A loading at centromeres is self-templated in the zygotic divisions. Therefore, the current proposed function for paternal CENP-A inheritance in flies is that is serves as a quantitative determinant of transgenerational centromere maintenance.

With respect to a specific function for paternal CENP-A, another species of fruit fly *D. virilis* possesses two CENP-A paralogs, namely *Cid1* and *Cid5* (Kursel et al. 2021). Somatic cells in this species express only *Cid1* whereas germline cells express both paralogs. However, there is a subsequent mutually exclusive retention of one chosen paralog in eggs and in sperm. In male meiosis, Cid1 is removed leaving Cid5 on mature sperm whereas in female meiosis, Cid5 is removed and only the somatic Cid1 is present in the mature oocyte. After fertilisation, Cid5 is absent from the early embryo and appears to be replaced with maternally derived Cid1 during protamine-to-histone exchange. These findings suggest a sperm-specific function for Cid5. CENP-A variants have been identified in mammals, for example in the bovine genome (Li and Huang 2008), but potential functional specifications have not yet been explored.

## Sperm CENP-A inheritance in mouse

The inheritance and potential functional importance of sperm CENP-A in mammals is only recently coming to the fore (Fig. 1). In 2022, Das et al. generated hemizygous *Cenpa*^*+/−*^ mice expressing half the CENP-A protein level of wild-type controls (Das et al. 2022). To study CENP-A inheritance across generations, the authors performed a cross between *Cenpa*^*+/−*^ parents and examined CENP-A levels in offspring. They noted a recovery in centromeric CENP-A levels in germ cells of female *Cenpa*^*+/+*^ littermates and a partial recovery in male offspring. Therefore, there is evidence for both compensatory CENP-A loading and epigenetic memory of CENP-A level in mouse. This contrasts with the epigenetic memory of paternal CENP-A reported in flies (Raychaudhuri et al. 2012) that could not be compensated in the embryo.

A series of reciprocal crosses between wild type and *Cenpa*^*+/−*^ parents went on to identify that the recovery to normal CENP-A levels was dependent on the maternal genotype. Weakened centromeres persist in the male germline of progeny produced by female hemizygotes, irrespective of the father's genotype. Yet, paternal centromeres completely recover in the progeny of wild type mothers. Another observation is that maternal *Cenpa*^*+/−*^ hemizygotes have reduced litter sizes, indicating compromised reproductive fitness as a functional consequence of weakened centromeres with reduced CENP-A. Taken together, findings from this study highlight CENP-A as a maternal effect gene in the determination of centromere strength in mice (Das et al. 2022).

In the same study (Das et al. 2022), the authors made a second unexpected set of observations. They measured an inherent asymmetry in parental CENP-A levels even in wild type zygotes; maternal centromeres contain approximately twofold more CENP-A than paternal centromeres. This asymmetry may reflect a loss of CENP-A from sperm in protamine-to-histone exchange or excess loading in the oocyte. Despite the initial differences, CENP-A distribution is equalised among paternal and maternal homologs by the 4-cell embryonic stage, after the time of ZGA in mouse. This indicates that an equalisation step is likely necessary to prevent the existence of strong and weak centromeres within the same cell that might need to compete for transmission, comparable to centromere drive observed in meiosis (Lampson and Black 2017; Akera et al. 2019). It is not known whether such an asymmetry exists in humans or other mammals in which ZGA occurs later than in mouse or whether any parental CENP-A equalisation events are necessary.

## Sperm centromeres: the next 40 years?

Studies with CREST serum in the 1980’s have been instrumental in the discovery of CENP-A and in the initial detection of centromeres in mature sperm nuclei (Fig. 1). However, precisely which centromere proteins are present on mature sperm remains to be determined. In *Drosophila*, CENP-A is exclusively retained, given that the only other CCAN protein in this species, CENP-C, is removed before spermiogenesis (Dunleavy et al. 2012; Raychaudhuri et al. 2012). In bulls, neither CENP-B nor -C was detected on sperm (Palmer et al. 1990), but these proteins were also not detected in the calf thymus, suggesting that human CREST did not recognise the bovine antigens. Western analysis using monoclonal antibodies has recently confirmed the exclusive retention of CENP-A in mouse sperm, indicating the absence of CENP-B and -C (Manske et al. 2022). Confirmation of this finding in human sperm will drive future studies into the transgenerational inheritance of paternal CENP-A in early embryogenesis and beyond.

In addition to determining precisely which centromeric proteins are retained on mature sperm, we also need to understand why and how CENP-A is maintained. Studies in flies have effectively addressed the functional importance of CENP-A maintenance on sperm for early embryo development (Raychaudhuri et al. 2012). Whether sperm CENP-A is essential for paternal chromosome function in the developing mammalian embryo is not clear yet. To understand mechanistically how centromeres are maintained, investigations into CENP-A stability and dynamics through the protamination process are needed. For instance, we do not know if CENP-A occupies the same DNA sequences before and after protamine exchange, or even if it exists in its octameric nucleosome form, in a tetramer with histone H4, or as the sole remaining histone. It is also unexplored how centromeric chromatin is organised in the context of DNA wrapped in protamine-bound toroidal structures (Fig. 2). Mechanisms of CENP-A retention on sperm might include specific histone posttranslational modifications or unique and protective interactions with other unknown proteins, which could be addressed through CENP-A purification and mass spectrometry studies. Indeed, Del Mazo et al. (1986) speculated some time ago that phosphorylation could be one of the mechanisms involved in the sperm chromatin packaging and thus influencing antigen recognition with CREST serum. Interestingly, studies performing immunofluorescence microscopy with anti-CENP-A antibodies in mouse gametes use lambda phosphatase in protocols, increasing accessibility of the antibody to the antigen (Das et al. 2022). Understanding the mechanism of CENP-A maintenance could also contribute to finding how and when other CCAN components are removed during spermatogenesis. Currently we do not know whether this is coupled to the removal of canonical histones, or to protamination, or if individual CCAN proteins play roles at distinct stages of spermatogenesis in mammals. It is also important to consider the contribution of centromeric DNA to functional centromere maintenance. Determining whether the typically hypomethylated region associated with CENP-A occupancy, called the centromere dip region (CDR) (Altemose et al. 2022a) is precisely maintained through spermiogenesis and inherited in mature sperm will be of particular interest.

Deciphering the mechanism of CENP-A maintenance is linked with understanding the overall organisation of centromeres within sperm nuclei, which may contribute to the shape of the sperm head in mammals. This is important as in human and cattle, scoring sperm head morphology is the standard procedure used for fertility prediction that commonly reflects defects in protamination or chromatin status (Simon et al. 2011; Utsuno et al. 2014; Boe-Hansen et al. 2018). It is possible that aberrations in centromere structure are reflected in defective nuclear morphologies too. In *Drosophila melanogaster* sperm, the four centromeric foci of the four chromosomes are spatially separated in the needle-shaped haploid nucleus (Dunleavy et al. 2012), while in *Drosophila virilis* the five centromeres cluster as a single focus located at one end of the sperm nucleus (Kursel et al. 2021). In mammalian sperm, centromeres appear to cluster centrally forming chromocenters (Del Mazo et al. 1986; Palmer et al. 1990; Zalensky et al. 1993). This type of packaging could serve to protect centromeres from DNA damage, as sperm pass through different environments prior to reaching the oocyte in the female fallopian tubes. Further Fluorescent in Situ Hybridisation (FISH)-based mapping studies of centromeric DNA are needed to determine the overall spatial organisation and packaging of centromeres throughout protamination and in mature sperm.

Finally, it is important to consider that disruptions to meiotic chromosome segregation are frequently linked to infertility and could potentially be related to centromere or CENP-A defects in sperm. Pioneering investigations in preimplantation human embryos donated from in vitro fertilisation clinics are critical for continued progress in this area. The development of human induced pluripotent stem cell technologies could also hold the key to understanding centromere dynamics and function in early human development. The use of genome editing in suitable animal models will continue to provide insights applicable to human biology. Genetic approaches can be coupled with super resolution imaging and expansion microscopy techniques that might overcome nuclear compaction to visualise centromere structure in sperm at the highest possible resolution. Approaches can be informed by complete Telomere-to-Telomere (T2T) centromere assemblies now available for multiple species. For instance, the long-read, single-molecule method DiMeLo-seq (Altemose et al. 2022b), could precisely map the position of CENP-A nucleosomes in healthy or abnormal sperm. After 40 years of studying CENP-A largely in somatic cells, researchers are now armed with detailed knowledge of its molecular, biochemical and structural properties. The field is now well positioned to uncover CENP-A dynamics and function in the germline, including the mechanisms by which it is maintained in sperm and to assess its transgenerational importance in fertilisation and early development.

## Data Availability

No datasets were generated or analysed during the current study.
